# The Model System *Saccharomyces cerevisiae* Versus Emerging Non-Model Yeasts for the Production of Biofuels

**DOI:** 10.3390/life10110299

**Published:** 2020-11-21

**Authors:** Maria Priscila Lacerda, Eun Joong Oh, Carrie Eckert

**Affiliations:** 1 Renewable and Sustainable Energy Institute (RASEI), University of Colorado, Boulder, CO 80303, USA; mariapriscila.lacerda@colorado.edu; 2 Department of Food Science, Purdue University, West Lafayette, IN 47907, USA; Eun.Oh@colorado.edu; 3 National Renewable Energy Laboratory (NREL), Biosciences Center, Golden, CO 80401, USA

**Keywords:** yeast, synthetic biology, biofuels, bioproducts, metabolic engineering

## Abstract

Microorganisms are effective platforms for the production of a variety of chemicals including biofuels, commodity chemicals, polymers and other natural products. However, deep cellular understanding is required for improvement of current biofuel cell factories to truly transform the Bioeconomy. Modifications in microbial metabolic pathways and increased resistance to various types of stress caused by the production of these chemicals are crucial in the generation of robust and efficient production hosts. Recent advances in systems and synthetic biology provide new tools for metabolic engineering to design strategies and construct optimal biocatalysts for the sustainable production of desired chemicals, especially in the case of ethanol and fatty acid production. Yeast is an efficient producer of bioethanol and most of the available synthetic biology tools have been developed for the industrial yeast *Saccharomyces cerevisiae*. Non-conventional yeast systems have several advantageous characteristics that are not easily engineered such as ethanol tolerance, low pH tolerance, thermotolerance, inhibitor tolerance, genetic diversity and so forth. Currently, synthetic biology is still in its initial steps for studies in non-conventional yeasts such as *Yarrowia lipolytica*, *Kluyveromyces marxianus*, *Issatchenkia orientalis* and *Pichia pastoris*. Therefore, the development and application of advanced synthetic engineering tools must also focus on these underexploited, non-conventional yeast species. Herein, we review the basic synthetic biology tools that can be applied to the standard *S. cerevisiae* model strain, as well as those that have been developed for non-conventional yeasts. In addition, we will discuss the recent advances employed to develop non-conventional yeast strains that are efficient for the production of a variety of chemicals through the use of metabolic engineering and synthetic biology.

## 1. Introduction

The production of biofuels and chemical bioproducts utilizing live cells and thus their enzymatic conversion pathways, requires less energy than other conversion processes. Therefore, microbial factories are increasingly being developed through the engineering of metabolic pathways and the redirection of carbon towards desired products [1]. The main challenge for the commercialization of biofuels and other chemicals is the gap in the path between the laboratory and the commercial market, mainly due to the fact that often the engineered strains do not fit commercialization needs [2].

Optimizing native gene expression and expression of non-native genes and pathways enables the engineering of microbes with new catalytic activities or improvements of native phenotypes such as tolerance to stress conditions [2,3]. Currently, metabolic engineering allows for a systematic workflow for designing more robust and efficient cell factories. Such a process is characterized mainly by manipulating the expression of pathway genes, balancing cofactors, eliminating by-products and increasing the supply of precursors, among other possible approaches [4]. Metabolic engineering integrates biological information underlying graphical and mathematical representations of metabolic flows, mainly in order to search for cellular functions that can improve the production of a desired compound or the development of a desired characteristic [5]. Recently, advanced sequencing technologies have enabled rapid analysis of genomic variations that lead to desired phenotypes [6]. In addition the development of new genome editing tools, such as CRISPR/Cas systems, have facilitated and increased the speed of construction of microbial cell factories [4]. Such efforts have paved the way for the engineering of higher efficiency production of biologically derived chemicals.

**Figure 1:**
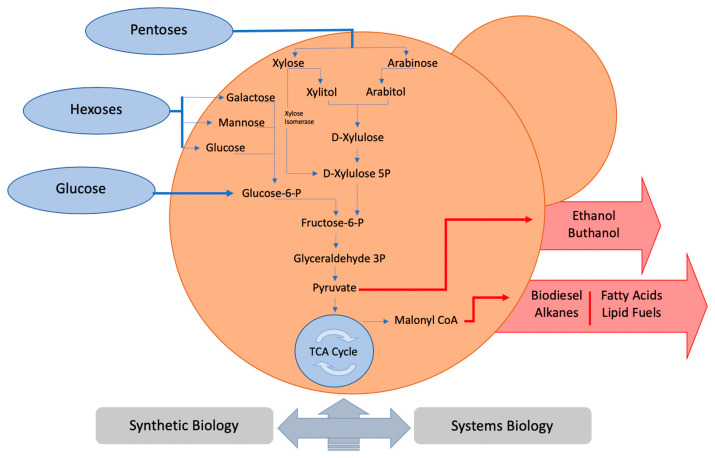
Yeast as a cell factory platform. Using systems biology and synthetic biology yeast metabolism can be engineered to produce a wide range of different chemicals.

With more than a thousand unique species already identified, yeasts are one of the most widely studied microorganisms. For thousands of years, these species have been associated with the production of fermented drinks [7]. The budding yeast *S. cerevisiae* has long been the favorite organism used as a cell factory in the commercial production of biologically-based chemicals, mainly due to its ancient use in classical industrial applications (wine and beer production) and our vast physiological and genetic knowledge of this organism [8]. A scheme representing different substrate assimilation routes is illustrated in the Figure 1. The efficient conversion of carbon substrates such as glucose, different types of hexoses and pentoses is processed through hydrolytic enzymes and transporters in yeast. These metabolic pathways lead to the production of the most important products in the biofuel industry, such as ethanol, butanol, alkanes and fatty acids.

Its success as a model organism is mainly due to the high degree of conservation of important cellular processes (autophagy, protein translocation and secretion, cell division, heat shock, protein folding and chaperone functions) and the availability of genetic engineering tools. In addition to its classic application in the production of wine and beer, its use has also extended to the production of bioethanol, aimed at the production of transport fuels. In 2017, the United States produced more than 60 billion liters, followed by Brazil with production of about 30 billion liters, with the rest of the world producing about 15 billion liters [5].

Despite the ease of engineering, *S. cerevisiae* has disadvantages, such as the inability to consume more economical and sustainable substrates such as xylose, arabinose and glycerol. Additionally, *S. cerevisiae* is not able to tolerate high temperatures (>34 °C) and low external pH (<3) [9], so its application in the production of second-generation biofuels has been limited. Non-conventional yeasts such as *Yarrowia lipolytica, Kluyveromyces marxianus, Issatchenkia orientalis and Pichia pastoris* have many physiological, metabolic and regulatory advantages that could overcome these limitations [3,10,11].

While most reviews in the literature addressing the engineering and characteristics of yeast for biofuel production focus on the model industrial yeast *S. cerevisiae* [12,13,14,15,16], there are fewer that have highlighted updated studies of non-conventional yeasts [3,17,18,19]. Here, we focus both on the advanced genome editing tools currently available for *S. cerevisiae and* their adaptation for use in non-model yeast systems to further improve the range and scale of industries for the biological production of chemicals using yeast platforms.

## 2. Conventional and Non-Conventional Yeast Bioproduction Systems

### 2.1. S. Cerevisiae for the Production of Chemicals Beyond Ethanol

As the first eukaryote to have its genome sequenced [20] and the ease in ability to genetically manipulate this organism, *S. cerevisiae* has been widely utilized as a model organism for basic science as well as for the production of biochemicals and heterologous expression of proteins [21,22]. Systems biology and synthetic biology tools are widely available for this organism and with the application of detailed mathematical models, the generated libraries can be analyzed through the omics sciences [23,24]. The Saccharomyces Genome Database (SGD; http://www.yeastgenome.org/), is a major scientific database that gathers genetic information from the yeast *S. cerevisiae*. This tool has developed several new features that allow to compare and integrate detailed information about individual genes throughout the genome, allowing to connect genetic groups with common characteristics [25]. This allows to expand the knowledge of certain biological behaviors and genetic regulation [23,24].

The potential of *S. cerevisiae* as a versatile host for metabolic engineering has been successfully demonstrated for the synthesis of a variety of molecules [22,26]. For production of these chemicals economically at industrial scales, it is necessary to optimize the fermentation titers, rates and yields (TRY). This process involves optimization of metabolic pathways to increase flux to the product of interest [5], as well as overcoming possible toxicity of the final product (or pathway intermediates) to the host organism [27].

As mentioned above, one of the main disadvantages of *S. cerevisiae* is its inability to tolerate important stress factors during the production of second-generation biofuels. In Table 1 a summary of the resistance factors of the species discussed is presented. One of the disadvantages of *S. cerevisiae* is also the lack of several metabolites of short-chain acyl-CoA that are necessary in the biosynthesis of economically important compounds, such as organic acids and linear alcohols [28]. These compounds can be derived from fatty acid biosynthesis and are promising alternatives to petroleum-based fuels [29]. Bioalkane is another targeted product because it can be used directly as biofuels and is high in energy density. The metabolic flow is limited mainly by the fact that most of the biosynthetic pathways use intermediate lipid biosynthesis strongly regulated acyl-CoA or fatty acyl transporter protein (ACP). Free fatty acids (FFA) can be accumulated and used for the biosynthesis of alkanes and fatty alcohols through the formation of a fatty aldehyde intermediate [30]. Foo et al. [31] expressed a cyanobacterial aldehyde deformylating oxygenase to convert free fatty acids into alkanes. In addition, Krink-Koutsoubelis et al. [26] introduced biosynthetic metabolic pathways in five different acyl-CoA esters, demonstrating heterologous expression of the alternative biosynthetic isovaleryl (AIB) pathway from *Myxobacterium xanthus*, as well as the first report of production of n-butyryl-CoA and n-hexanoyl-CoA in *S. cerevisiae*.

The production of terpenoids and isoprenoids using *S. cerevisiae* as a cell factory has attracted great interest, mainly because they are currently chemically synthesized, which adds high cost to their production. This important class of secondary metabolites has attractive industrial applications, such as flavor and fragrance additives in the food and cosmetics industry, medicines and biofuels. The biosynthesis of terpenoids in *S. cerevisiae* uses two C5 precursors: isopentenyl diphosphate (IPP) and dimethylallyl diphosphate (DMAPP). In yeasts and mammals, acetyl-CoA is converted into mevalonic acid leading to the production of IPP. Pre-transferase enzymes act on IPP synthesizing building blocks such as geranyl pyrophosphate (GPP); farnesyl pyrophosphate (FPP); and geranyl pyrophosphate (GGPP). FPP is usually the most common and desired product of the pathway that is used for sterol biosynthesis [54,55].

Isobutanol is also a promising chemical production platform for *S. cerevisiae* but its level of natural biosynthesis in yeast *S. cerevisiae* is very low. By blocking all competing unproductive pathways by gene deletions and expressing the cytosolic pathway of isobutanol, it was possible to produce 57 times more isobutanol when compared to the studied parent strain. Using isobutanol production pathways located in mitochondria, the respiratory response induced by xylose can be better used in engineered yeasts to use this substrate. Lane et al. [56] employed different isobutanol biosynthesis pathways targeted at mitochondria, increasing the isobutanol production titers by up to 23 times and reaching 2.6 g/L of isobutanol in fermentations in flasks and bioreactors. The results indicate that the capacity of the isobutanol pathways is still unable to compete with the ethanol and glycerol biosynthesis pathways for example but these results emphasize the need for more metabolic studies for the optimization of viable strains for industrial application [57].

### 2.2. Y. lipolytica, K. marxianus, I. orientalis and P. pastoris Cell Factories for the Production of Chemicals

#### 2.2.1. Yarrowia Lipolytica

Although the yeast *S. cerevisiae* is the most industrially applied and most studied in the field of yeast engineering, its efficient performance in the production of ethanol occurs under aerobic growth, which defines it as “Crabtree-positive” [58]. However, this characteristic is considered an obstacle during production, as it results in low yields of some metabolites including organic acids, diols and diamines [59]. The non-conventional yeast *Y. lipolytica* is not able to produce ethanol from the moment glucose is supplied to respiring cells, which defines it as “Crabtree-negative” [60,61]. This yeast is capable of consuming a variety of carbon sources including oils and hydrocarbons and has been engineered to produce fatty acid methyl esters with titers up to 99 g/L [62]. Strains grown on substrates derived from sustainable lignocellulosic raw materials have been developed for the production of compounds based on acyl-thioester [63]. *Y. lipolytica* produces various types of lipases that can be used as biocatalysts and is able to accumulate lipids (more than 80% cell dry weight) that can be used as starting materials for the production of biodiesel [64]. The main focus of *Y. lipolytica* research so far has been to elucidate its metabolism involving the biosynthesis of fatty acids, use of hydrophobic substrate and lipid degradation, such as β-oxidation [63].

In addition to biodiesel as a product, Blazeck et al. [65] designed a metabolic pathway for the production of pentane in *Y. lipolytica*, a compound used mainly in variable mixtures of gasoline and aviation fuel. They used a soy lipoxygenase enzyme to separate linoleic acid into pentane and a tridecadienic acid as a by-product, producing pentane at ~5 mg/L. *Y. lipolytrica* has also been engineered to produce α-Farnesene, a terpenoid compound that can be utilized as a precursor to aviation fuel at 260 mg/L [66].

*Y. lipolytica* has shown great compatibility in large-scale bioprocesses, facilitating its use in the production of biofuels (biodiesel and bio-oil) [64]. Despite this, the industrial viability of the glucose to lipid conversion process is still not financially favorable. Increased dissolved oxygen levels in *Y. lipolytica* cultures leads to increased citrate concentrations [67], resulting in increased acetyl-CoA in the cytosol, a mechanism different from non-oleaginous yeasts, as they use the pyruvate dehydrogenase derivation route [68]. Metabolic engineering efforts are currently focused on (1) better secretion of fatty acids to facilitate better extraction and separation; (2) developing sources of nitrogen or phosphorus toxic to contaminant organisms to enable use of non-sterile media; and (3) development of strains able to assimilate low-cost substrates such as xylose, fructose and galactose [69]. In addition, the construction of synthetic promoters with a broad range of expression levels and tunability is an area of ongoing studies to further optimize flux to products [70].

#### 2.2.2. Kluyveromyces Marxianus

*K. marxianus* is also defined as a Crabtree negative (respiring) yeast and has advantages over other yeast species, mainly because it has greater thermal stability (>40 °C), low pH tolerance [71], a relatively fast growth rate and a highly resistant cell membrane [72]. Fermentative processes when carried out at high temperatures reduce cooling costs, in addition to reducing problems caused by contamination [46]. *K. marxianus* also has a high secretory capacity in relation to *S. cerevisiae*, due to properties such as appropriate glycosylation and strong signal peptides [73].

This strain has been viewed as an alternative to *S. cerevisiae* in 2nd generation ethanol processes, as it is able to naturally assimilate a variety of sugars in addition to glucose, such as pentose, hexose, arabinose, cellobiose, lactose and xylose, as well as some toxic compounds present in some sources of lignocellulosic biomass [74,75]. Pentjuss et al. [76] applied and experimentally validated a stoichiometric model of central metabolism linked to biomass, to evaluate the efficiency of carbon conversion of *K. marxianus*. Substrates such as lactose, glucose, inulin, xylose were evaluated. The modeling results suggested that the aeration control can optimize the product yield and distribution of the metabolic flow in *K. marxianus*. Gao et al. [77], aiming at a better understanding of ethanol production from inulin in *K. marxianus*, conducted experiments analyzed through RNA-seq, which allowed the identification of genes associated with ethanol metabolism. It was observed through the regulation of global expression that the increased expression of inulinase and the low activity of the gene related to autophagy, ATG8, guaranteed fast and ideal fermentation processes.

Although *K. marxianus* can effectively transport and metabolize pentoses via the aldose reductase pathway, it is still necessary to modify the metabolism of wild type strains for the production of ethanol from pentoses. The strategies used in the development of *K. marxianus* strains to improve their ability to ferment pentoses are generally mirrored in studies carried out on *S.* cerevisiae [78]. One of the first studies designed a strain capable of generating ethanol from xylose with a yield of 37%, by replacing the KmXYL1 gene with the Schefferomyces stipitis xylose reductase gene, which has a double cofactor specificity [79].

It is known that *K. marxianus* grows well, replicating more quickly in aerobic conditions without fermentation to ethanol, reaching production of 5.1 g/L of ethanol from 10 g/L of glucose and a growth rate of 0.58–0.63 h^−1^ even at a temperature of 45 °C [80]. Due to its efficient ethanol production capacity and thermotolerance, it has been applied in high temperature fermentation technologies (HTF) for ethanol production. Such fermentation process allows for the product to be recovered and concentrated using low pressure distillation, which reduces production costs, as well as cooling and enzymatic hydrolysis costs and additionally prevents bacterial contamination and improves the simultaneous saccharification and fermentation (SSF) performance [81,82].

The general metabolism of *K. marxianus* in relation to glycolysis and the tricarboxylic acid (TCA) cycle and the dynamics of the corresponding genetic controls are still underexplored [59]. *K. marxianus* has been genetically manipulated in order to increase its ethanol production efficiencies. Despite having excellent characteristics for bioprocesses, its capacity to resist ethanol is relatively low, being able to support only a maximum of 6% (*v*/*v*) of ethanol. This low ethanol tolerance has the consequence of low ethanol yield, being the main obstacle to practical application of this yeast in the industry [83,84]. Diniz et al. compared the *K. marxianus* transcriptomes in adaptive evolution in 6% (*v*/*v*) ethanol using lactose as a carbon source. As a result, they obtained a decrease in the central metabolic flow and the biosynthesis of fatty acids. Li and colleagues examined random mutagenesis library of the TATA-binding protein of *K. marxianus*, Spt15, which facilitates the differential expression of hundreds of genes that act as an interconnected network for the ethanol tolerance phenotype. In this study, a strain with a maximum tolerance of 5% (*v*/*v*) of ethanol was obtained, while the original wild type strain had a tolerance of only 2% (*v*/*v*). With a metabolism similar to *S. cerevisiae*, the ethanol tolerance of *K. marxianus* also has a genetic and metabolic complexity that makes efficient strain engineering difficult, thus requiring greater efforts to understand its molecular base involved in resistance mechanisms to factors of stress [85]. In a study conducted by Fu et al. [86] fermentations at high temperatures were conducted under different stress conditions including ethanol, acetic acid and reactive oxygen species (ROS) followed by RNA sequencing (RNA-Seq) and metabolite data analysis. It was observed that ergosterol production increased in response to ethanol stress. The regulation of genes related to peroxidase activity, the assembly of the iron-sulfur cluster and the binding to flavin mononucleotide (FMN) were negatively regulated, while the genes associated with DNA repair and the lipid composition of plasma were regulated positively.

The key element for using *K. marxianus* in industrial applications is the development of improved genetic tools to more quickly and effectively manipulate the genome. There are efforts to develop these tools in *K. marxianus*, mainly aiming at the modification of metabolic pathways [78,87].

#### 2.2.3. Issatchenkia Orientalis

First described as a wine yeast, the yeast *I. orientalis*—also known as *Pichia kudriavzevii* [88]—has several resistance characteristics, such as thermal tolerance, low pH tolerance, salt tolerance and high tolerance towards multiple organic acids to produce ethanol. *I. orientalis* also has shown great potential in the production of succinic acid and bioethanol [89,90].

*I. orientalis* can produce ethanol under various stress conditions including high temperatures (42 °C) and high concentrations of Na_2_SO_4_ at pH 2.0. Production of d-xylonate or succinic acid have also been demonstrated at a low pH of 3 [89,91,92,93]. This ability to grow at low pH makes it a perfect platform for the production of organic acids. The expression of native or heterologous enzymes have been demonstrated to increase production of some acids by fermentative processes [94]. Xiao et al. [89] improved the reductive cycle of TCA to produce succinic acid through genetic engineering of *I. orientalis* SD108. The projected strain achieved a production of 11.63 g/L of succinic acid, with a yield of 0.12 g·g^−1^ and productivity of 0.11 g/L. The production of succinic acid using *I. orientalis* has also been demonstrated industrially by companies such as BioAmber, DSM and Roquete. Bioamber designed a strain capable of producing 89 g/L of succinate at a production rate of 0.93 g/L/h. DSM and Roquette through a partnership sell Biosuccinum through bioprocesses with the strain SUC-297 that produces 43 g/L of succinic acid [95].

The genetic manipulation of this organism presents the same difficulties in relation to other non-conventional yeasts due to the lack of efficient genetic editing tools specific to this organism [96]. Cao et al. [96] recently developed a set of genetic tools for *I. orientalis* to build a path for the functional use of xylose. The CEN-L sequence identified through functional screening was able to improve the expression of the target genes. The development of this plasmid enabled a stable CRISPR/Cas9 system for introduction of multiplexed gene deletions. These technologies are fundamental for future metabolic engineering of *I. orientalis* [97].

#### 2.2.4. Pichia Pastoris

*P. pastoris* is a Crabtree-negative methylotrophic organism used industrially as a host to produce recombinant proteins in the pharmaceutical industry and in the production of higher alcohols and corresponding acetate esters [98,99]. The strength of *P. pastoris* for heterologous expression of cellulolytic and hemicellulolytic enzymes makes it a really attractive yeast for industrial bioprocesses [98].

*P. pastoris* has been used as a cell biocatalyst for the production of biodiesel due to superficial display of lipases. Another advantage is that its cultivation can be carried out at high cell densities in a medium containing crude glycerol as a carbon source, a by-product of biodiesel production [100]. Crude glycerol contains substances such as glycerol, methanol, soap, some salts and water, in addition to other impurities [101]. For most microorganisms, methanol is often toxic but *P. pastoris* is capable of metabolizing this compound [102].

Enzymes such as lipases have been studied in catalytic processes in the production of biodiesel [103]. The use of lipases reduces the cost of production, since its catalytic activity requires mild conditions and, as a consequence, less energy consumption compared to chemical methods [104]. Studies have shown that an immobilized lipase from *Streptomyces sp*. can be expressed heterologously in *P. pastoris* and this system can be applied for the production of biodiesel by adding a methanol stage in systems without the use of solvent [105]. Liu et al. [106] used *P. pastoris* as a whole cell biocatalyst (WCB) with overexpression for improved thermostability. The optimum conditions studied led to a fatty acid methyl ester (FAME) yield of 60.7%, with a 47.3% yield after 4 batch cycles without glycerol generation. This indicates that the catalyst system used is promising and potentially economical for the production of biodiesel.

Expression and secretion of enzymes that aid sugar release and utilization are also of interest to improve production processes. Glucuronoesterase (GE) is a carbohydrate esterase that is able to cleave alkaline bonds of lignin-carbohydrate complexes under acidic pH conditions [107]. The removal of branches of glycuronic acid from hemicellulose can improve the release of fermentable sugars. Conacher et al. [108] reported the production and secretion of GE at high yields through constitutive expression in *P. pastoris*. Li et al. [109] expressed *Rhizomucor miehei*-derived β-mannanase (mRmMan5A), reaching a high level of expression (79,680 U m/L). This expression rate makes the process highly economical for industrial applications. MRmMan5A was used to hydrolyze a substrate for the production of manno-oligosaccharide. Approximately 80% of mannan in the substrate was converted into manno-oligosaccharides. These results suggest an extremely effective β-mannanase for the bioconversion of biomass rich in mannan.

Promoter engineering has increased its expression capacity in addition to improving the ability to metabolically engineer *P. pastoris*. With the advancement of new genome engineering technologies including CRISPR/Cas9 genome editing to optimize strains, the versatility and competitiveness of this species can be further improved in the expression of enzymes for the production of second-generation ethanol [110] and other desired chemicals.

## 3. Advancing Technologies for Optimization of Production Pathways and Host

**Figure 2:**
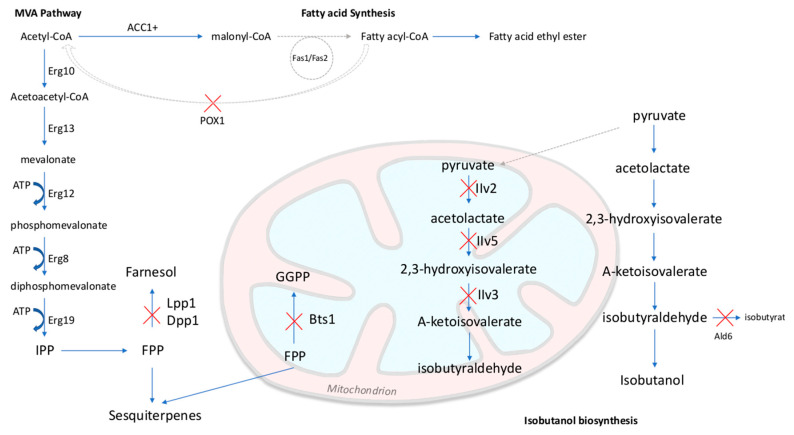
Sesquiterpenoid, Fatty acid ethyl ester and Isobutanol biosynthesis in *S. cerevisiae*. Upper cases: general enzyme types; lower cases: specific proteins. ATP = adenosine triphosphate; IPP = isopentenyl diphosphate; FPP = farnesyl diphosphate; GGPP = geranylgeranyl diphosphate; MVA = mevalonate; ACC = acetyl-CoA carboxylase. + = Overexpressed genes. Red crosses: enzymatic steps eliminated by gene deletion.

The data obtained through systems biology and synthetic biology applied to the development of microorganisms can decrease the cost of producing desired chemicals simply by reducing the number of experiments necessary to optimize the system through the utilization of omics data to guide design to control expression, redox and flux to increase titers towards the chemical of interest [3,111]. In addition, feedstock utilization and increased resistance to metabolic intermediates, by-products, final products and feedstock materials are also necessary to optimize production. The design of yeast cell factories can be accomplished through the use of two approaches, rational metabolic engineering and reverse metabolic engineering. Rational metabolic engineering is based on the construction of strains by genetic engineering, using physiological, biochemical and genetic data of the strain. Reverse metabolic engineering is based on the selection of cellular systems that present cellular phenotypes similar to the one desired, using comparative analysis to identify genetic differences expressed between the studied systems [24]. These engineering strategies rely on our ability to quickly and efficiently modify strains genetically. These genetic manipulations are enabled by the development of advanced genetic engineering tools including plasmids, tunable promoters, CRISPR-Cas systems for genome editing and regulation and other tools to enable high throughput methods. Below we describe these systems available for model and non-model yeasts. A scheme representing possible pathways to be modified regarding sesquiterpenoid, fatty acid ethyl ester and isobutanol production is illustrated in the Figure 2.

### 3.1. Classical Genetics Tools and Emerging Technologies

Beyond standard homologous recombination, next generation genetic manipulation tools such as zinc finger nucleases (ZFNs) and transcription activator effector nucleases (TALENS) were some of the first approaches used for accurate genome editing. Zinc finger nucleases (ZFNs) are hybrid restriction enzymes comprised of a customizable zinc-finger protein DNA-binding domain, which is fused to the FokI endonuclease cleavage domain. ZFN induce double-strand breaks that through cellular DNA repair processes have their genes replaced and targeted at high frequencies. Many different sequences can be attacked allowing new assemblies of ZFs. For this reason, dimerization becomes an advantage where cleavage does not occur at single binding sites and requires that the fingers have adequate specificity [112].

TALENs are also customizable restriction enzymes as well as zinc finger nucleases (ZNFs), consisting of a fused DNA-binding domain to a non-specific nuclease domain. Its DNA binding domain contains several highly conserved repeating sequences consisting of up to 35 amino acids that bind to a single DNA base with specificity for binding to two unconserved amino acids. A challenge in using this approach is the need to build plasmids capable of encoding long repeating arrays [113]. TALENs have been successfully used in *S. cerevisiae* for the overproduction of fatty acids [114] and have also been utilized in *Y. lipolytica* [115], demonstrating that this technology has potential to be further explored for engineering in non-conventional yeasts.

These methodologies were developed to specifically cleave DNA sequences; however, they are not advantageous for editing multiple targets and involve a more laborious and time-consuming construction of DNA-binding proteins [11]. Methodologies using CRISPR-Cas systems have revolutionized microbial genetic engineering because they are more efficient than traditional techniques, with low cost and quick analysis. This system enables binding of the Cas9 nuclease to a target DNA sequence through a guide RNA (gRNA). The Cas9 protein breaks the double strand (DSB) into three nucleotides upstream of the PAM site (adjacent protospacer motif). The introduction of the DSB must be repaired by non-homologous end joining (NHEJ) or alternatively, homology-directed repair, to prevent the death of the host [18,116].

The CRISPR-Cas based editing systems can be utilized for high throughput, multiplex genome editing technologies for genotype-phenotype discovery. Array-based oligo technologies enable creating hundreds of thousands of cassettes containing a donor template and a genome-targeting gRNA and a pooled plasmid library generates designed mutations in parallel. After CRISPR/Cas9 genome editing, each plasmid can be served as a genetic barcode and the frequency of each designed mutation can be tracked by next-generation sequencing (NGS). This approach was applied to improve ethanol tolerance and production in *S. cerevisiae* by perturbing 25 regulatory genes with 43,020 mutations [117]. Also, genetic parts encoding overexpression and knockdown mutations of >90% yeast genes were iteratively integrated into the repetitive genomic sequences in a modular manner based on multiplex integration by CRISPR/Cas9. This approach with the aid of robotic automation allowed functional mapping and multiplex optimization on a genome scale for diverse phenotypes such as acetic acid tolerance [118]. A unique advantage of the CRISPR/Cas9 system is that multiple gRNAs can be combined with Cas9, achieving efficient multiplex genome editing. The system could accelerate future genome-scale engineering in polyploid industrial strains [118]. Furthermore, the use of lab-on-chip, ultra-low volume nanoreactors allows for high index of selection of variants [119]. If the variant library is pooled and a growth selection to enrich for improved variants is not available for the desired phenotype, other methods for high throughput screening or selection are required.

The CRISPR/Cas9 technology has been applied to non-model yeasts, based on the foundation of *S. cerevisiae* systems. Since little information on CRISPR/Cas9 toolbox such as RNA polymerase III promoter for Cas9/gRNA expression and autonomously replicating sequence (ARS) for plasmid expression in host strains is available for several yeasts, the implementation of CRISPR/Cas9 is closely related to the development of optimized expression system [97,120,121]. Synthetic promoters based on non-coding RNA and t-RNA promoters were used to express Cas9 and gRNA in a single episomal plasmid, enabling *PEX10* targeting efficiency up to 92% [122]. Similarly, CRISPR/Cas9 mediated disruption efficiency was found to be promoter-dependent in *K. marxianus*. Among the design of native and hybrid RNA polymerase III promoters for gRNA expression, the RPR1-tRNA^Gly^ hybrid promoter achieved the highest knockout efficiencies [123]. Recently, Tran et al. reported that ARS from *S. cerevisiae* was functional for plasmid replication in *I. orientalis* and the resulting episomal plasmid with a hybrid RPR’1-tRNA^Leu^ promoter for gRNA expression could attain greater than 97% gene disruption efficiency for various gene targets. Also, the platform was expanded to double and triple gene knockouts, resulting in disruption efficiencies of 90% and 47%, respectively [97]. Weninger et al. examined the optimized CRISPR/Cas9 systems in *P. pastoris*. They tested 95 combinations where they varied codon optimized Cas9 variants, gRNA sequences and ribozyme-coupled RNA Polymerase III and RNA Polymerase II promoters for expression of gRNAs and RNA Polymerase II promoters for Cas9 expression. Only 6 of 95 constructs were functional for efficient genome editing, suggesting that optimal conditions for efficient CRISPR/Cas9 function for *P. pastoris* had a very narrow range [124].

Another key tool in synthetic biology are biosensors to enable tunable control of systems or to act as a selection for key metabolites. Based on RNA aptamers or proteins linked to small molecules, biosensors are capable of eliciting a desired transcriptional or allosteric response. Its applications focus on notification of a certain metabolic state or even heterogeneity in the cellular response when coupled with regulation. Several biosensors have been successfully developed to regulate optimal gene expression, reducing inhibitory compounds or toxic proteins without the need for inducers. Its application has also been extended to control of pathways in response to metabolic switches [125].

The development of biosensor systems depends on the addition of sensor elements to achieve their functionality. For example, genes that encode extracellular receptors are controlled by an inducible promoter and coupled to an easily detectable output, such as fluorescence, luminescence, colorimetry or growth rate [126]. As example, Baumann et al. [127] developed a whole cell biosensor for quick and easy detection of Short- and medium-chain fatty acids (SMCFA). This biosensor was based on a yeast plasmid containing the PDC12 promoter responsive to SMCFA and coupled to GFP as the reporter gene. This sensor was able to detect hexanoic, heptanoic and octanoic acid, correlating strongly with the concentrations of octanoic acid and making it possible to screen the yield of SMCFA producers. Zhang et al. [128] developed the first genetically encoded biosensors for the metabolism of BCAA (branched chain amino acids) and the biosynthesis of BCHA (branched-chain higher alcohols) in *S. cerevisiae* aiming at the production of isobutanol and isopentanol. A transcriptional regulator of BCAA biosynthesis (Leu3p) was used to produce isobutanol. With some modifications it was possible to implement Leu3p in a different biosensor for isopentanol, another product of interest derived from BCAA. Also, light is an attractive source for the biosensor systems to control gene expression in yeast. A light-sensitive transcription factor from *Erythrobacter litoralis* (EL222) and its corresponding C120 promoter (PC120) were used to construct bidirectional gene circuits to either induce or repress genes of interest with light for microbial chemical production. The optogenetic regulation of metabolic pathways enabled optimizing engineered pathways and fermentation conditions using periodic light pulses [118].

Successful experiments with *S. cerevisiae* have boosted the application of CRISPR-Cas9 systems in various non-conventional yeasts, however it is necessary to overcome difficulties such as lack of stable plasmids, lack of native RNA promoters, limitation of the Cas9 NGG PAM motif and low homologous recombination (HR) efficiency [11]. Homologous recombination in non-conventional yeasts has become its main limitation in genetic engineering compared to *S. cerevisiae*, which has a high native capacity to perform homologous recombination [129,130].

CRISPR-Cas9 systems can help improve these yeasts as cell factories and increase the availability of bioproducts. Thus, a CRISPR multiple interference system (CRISPRi) was developed to redirect carbon flow from the central pathways in *K. marxianus* and efficiently targeted metabolic regulation increased the ethyl acetate titer by 3.8 times [131]. Applying another CRISPR approach, Schwartz et al. [132] used the activation of CRISPR (CRISPRa) in *Y. lipolytica* to positively regulate two β-glucosidases (BGL1 and BGL2), enabling this yeast to use cellobioses as the only carbon source.

CRISPR-Cas9 systems have been applied to overcome the challenges associated with previous methodologies. Still, the most significant issue required to improve the utility of CRISPR-Cas9 systems further is off-target effects. Potential off-target cleavage activity can bring unwanted sequence changes in the genome, resulting in genomic instability [133]. To reduce the off-target effects, designing gRNA with low homology to non-target genome sequences is essential. In silico tools have been widely applied to predict potential off-target sites for gRNA designs and avoid off-target binding [134,135,136]. Also, Cas nucleases can be engineered for higher targeting specificity with reduced off-target binding than wild-type nucleases. Liu et al. reported that Chimeric nucleases isolated from a synthetic library exhibited altered kinetic characteristics and enhanced editing specificity [137]. Rational engineering of the DNA binding region in Cas9 (eSpCas9) significantly reduced off-target effects, showing robust on-target cleavage activity [138]. The advances in designing gRNA and engineering Cas9 nucleases could expand the availability of efficient CRISPR-Cas systems for engineering non-model yeasts.

### 3.2. Systems Biology in Engineering Yeasts for Bioproduction

Systems biology has become the key tool for efforts in bioprocesses, bringing innovation in the understanding and development of biological pathways or processes in order to achieve the objectives of production on an industrial scale [139,140]. The development of powerful sequencing tools and techniques has enabled a vast amount of genome data, especially for model organisms. Through a data-driven approach, this data can be analyzed to achieve a vast understanding of the holistic form of cellular metabolism.

Bioinformatics has complemented systems biology with new computational and rational strategies, allowing for a comprehensive view of living information from the cellular environment as a result of omics analyses [139]. Unlike molecular biology that focuses on subsystems analyzed through in vivo and in vitro experiments, systems biology uses a holistic approach to study biological systems in a complete way, mainly through quantitative analysis [140]. Computational models can be used to structure and integrate -omics datasets in order to analyze in silico and globally the cellular metabolism. The main advantage of these models is the large amount of combinatorial genetic changes that can be tested without the need for individual laboratory tests and they can also predict which is the best route or metabolic path to be tested experimentally [141].

As a systems biology tool, genome scale metabolic models (GEMs) are characterized by connecting the omics sciences (genes, proteins and reactions) making possible metabolic and phenotypic predictions and representing valuable specific microorganism databases. Table 2 summarizes key characteristics of GEMs for the discussed yeasts. These GEMs have significantly developed the use of yeasts as platforms for integrating omics datasets and for in silico strain engineering [142]. The use of GEMs to generate phenotypic predictions on a genome scale, such as genetic knockout or amplification targets, for example, is mainly based on the application of various algorithms built through flow analysis [143]. In relation to genetic knockout targets, many computational tools have been used to identify new targets to increase the production of compounds. As examples of some algorithms using genetic knockout, MOMA was one of the first developed to increase the production of lycopene, l-valine, poly (lactic acid) and terpenoid. Optknock is another algorithm widely used in metabolic engineering to increase the production of 1,4-butanediol, for example. The OptSwap algorithm has also been widely applied, mainly for its additional function to optimize the specificities of cofactor oxidoreductases, making it possible to find new targets and monitor changes during the flow [144].

In relation to the identification of targets (up and down regulation) of gene amplification, algorithms based on analysis of flow response have been used for improving production. For example, OptReg and Optforce identify strategies combined in a comparative way between deletions and super-down expressions, for the optimization of hosts as MILP (Mixed-Integer Linear Programming) solutions [148,149,150]. Flux scanning based on enforced objective flux (FSEOF) [151] is an important and the most used technique based on FBA (Flux balance analysis) that identifies targets for overexpression, considering the status of wild type flow when identifying positive regulation targets as annotated genes for reactions whose flow is overexpressed in silico. Despite advances in the development of these algorithms, an accurate prediction of cellular phenotypes is not yet achieved. As a solution to this problem, GEMs have been integrated with the omics datasets to create more accurate models [144]. Computational forecasting has driven the development of new technologies to alter metabolic pathways that help in the production of biofuels, using knockouts or adding new functional enzymes. An example is the From Metabolite to Metabolite (FMM) software [152], it complements data from the kegg map to obtain a combined metabolic pathway [3].

The genetic modification of *S. cerevisiae* and non-conventional yeasts has been the main approach adopted in metabolic engineering for fermentation of sugar in ethanol in relation to second generation ethanol. The goal is to create or improve ways to use five-carbon sugars and ethanol synthesis in the same organism [153]. Although the yeast *S. cerevisiae* is an ideal host used in industrial fermentations, this yeast has no metabolic capacity for xylose or arabinose. *S. cerevisiae* has a putative xylose metabolic pathway; however, its level of expression is generally very low for using xylose as the sole carbon source. Therefore, this yeast needs further studies for effective genetic engineering of these pathways for the use of xylose and arabinose [154].

The engineering of *S. cerevisiae* strains to ferment with xylose has focused on the xylose reductase (XR) pathway (dependent on a cofactor) or xylose isomerase (XI) pathway (independent of cofactor). The XR route offers an advantage in terms of productivity of ethanol as a product, whereas the XI route in terms of product yield [155]. Controlling the expression of pentose phosphate pathway (PPP) genes is essential for the production of bioethanol. Overexpression of non-oxidative PPP genes (transaldolase (TAL1), transketolase (TKL1), ribose-5-phosphate ketol-isomerase (RKI1) and D-ribulose-5-phosphate 3-epimerase (RPE1)) are reported to increase the production of ethanol with xylose as the sole carbon source. However, the effects of exogenous expression of PPP genes are still uncertain as to xylose metabolism. The metabolic flow of each sugar component of the fermentation substrate may be related to xylose metabolism during ethanol production [156].

The optimization of roads can have several unexpected consequences, unbalancing the cellular system. These consequences generally lead to the accumulation of often toxic intermediates, formation of secondary metabolites, alteration in the metabolic load and, still, low yield of the product. In the last few years, new tools for optimization of pathways have become more economically viable in relation to commercial synthesis of DNA and genome editing systems in a multiplexed way. These technologies have allowed the use of libraries in which road elements are simultaneously diversified, facilitating combinatorial optimization of roads [157].

## 4. Conclusions and Future Directions

Synthetic biology has made it possible to control several cellular processes in order to obtain biofuels, the demand for which has increased in recent years. However, the metabolic engineering of cell factories still faces many limitations. The libraries of promoters and terminators, for example, still need to achieve greater amounts of sequence and functional diversity. Computational tools have driven the development of new hosts, facilitating the experimental design of new metabolic pathways and the understanding of genomic data.

New strategies and tools are continually progressing for engineering non-microbes, so that they can be optimized for real industrial applications. Such natural microbial genetic obstacles have been overcome with tools such as CRISPR/Cas systems and DNA assembly, improved computer models, automation and design by computer systems that quickly build and characterize the cellular genome. The vast availability of generated biological data has more rapidly developed data science and artificial intelligence strategies to suggest better strategies for genetic and metabolic engineering of cellular systems [158,159].

The simplicity in terms of regulation and the availability of excellent databases of the *S. cerevisiae* genome, in comparison to other yeasts, make this yeast the model organism even more used in research for biofuels. However, the development of new techniques of systems biology and synthetic biology to generate high cellular performance, has also made possible the genetic engineering of non-conventional yeasts, whose phenotypes of cellular resistance are of extreme interest in the fuel industry.

## Figures and Tables

**Table 1:** Summary of stress tolerance limits of all discussed species.

Organism	Glucose (*w/v*)	Salt	Temp	Ethanol (*v*/*v*)	5-HMF * (g/L)	pH
*S. cerevisiae*	40% [32]	0.4 M NaCl [33]	39 °C [34]	10% [35]	3.0 g [36]	3 [37]
*Y. lipolytica*	30% [38]	2 M NaCl [39]	35 °C [40]	5% [41]	2.0 g [42]	3.5 [43]
*K. marxianus*	40% [44]	1.5 M NaCl [45]	52 °C [7]	6% [46]	3.84 g [47]	3.5 [48]
*P. pastoris*	40% [49]	1.6 M NaCl [50]	41 °C [51]	6% [51]	-	3 [52]
*I. orientalis*	48% [7]	0.85 M NaCl [7]	45 °C [7]	10% [53]	7 g [7]	2 [37]

* 5-HMF: 5-hydroxymethylfurfural.

**Table 2:** Properties of key microbial genome-scale models.

Organism	No. of Genes	No. of Reactions	No. of Metabolites	No. of Compartments	Reference
*S. cerevisiae*	1147	3991	2691	14	[142]
*Y. lipolytica*	895	2002	1847	16	[145]
*K. marxianus*	996	1913	1531	-	[146]
*P. pastoris*	915	1423	899	7	[147]
*I. orientalis*	-	-	-	-	-
